# CCR10: a comprehensive review of its function, phylogeny, role in immune cell trafficking and disease pathogenesis

**DOI:** 10.3389/fimmu.2025.1599277

**Published:** 2025-09-19

**Authors:** Amanda Lopes Willuveit, Ana Carolina Buzzo Stefanini, Taís Matozo, Luciana Cavalheiro Marti

**Affiliations:** Hospital Israelita Albert Einstein, Experimental Research Department, São Paulo, Brazil

**Keywords:** chemokine, chemokine receptor, CCR10, CCL27, CCL28

## Abstract

CCR10, the latest classified receptor in the CC chemokine family, plays a critical role in tissue-specific immune responses, particularly in skin and mucosal immunity. By interacting with its ligands, CCL27 and CCL28, it regulates immune cell trafficking, contributing to homeostasis, wound healing, and mucosal defense. However, CCR10 has also been implicated in inflammatory disorders, autoimmune diseases, and cancer progression, where it may facilitate immune evasion and metastasis. Despite its dual roles, CCR10 represents a promising therapeutic target, potential applications in modulating immune responses for inflammatory diseases and oncology. A deeper understanding of its mechanisms and interactions could provide valuable insights into immune system regulation, disease progression and clinical relevance. This review explores CCR10’s molecular structure, biological functions, and potential for therapeutic intervention.

## Introduction

Chemokines are small polypeptides (8–14 kDa) that regulate immune homeostasis by balancing pro-inflammatory leukocyte recruitment with homeostatic migration (1, 2). They are crucial for immune cell movement, supporting development, maintenance, primary and memory immune responses, and homing to inflammation or disease sites (3). Chemokines are classified into two major subfamilies: CXC and CC, based on the positioning of two cysteine residues. CXC chemokines have an amino acid between them, whereas CC chemokines have adjacent cysteines (1). Their effects are mediated via G protein-coupled receptors (GPCRs), which typically bind multiple chemokines within the same subtype, ensuring specificity in signaling (4, 5).

CC-type chemokines (CCL1–CCL28) signal through receptors (CCR1–CCR10) (5, 6). Leukocytes express diverse chemokine receptor profiles, primarily GPCRs that, upon ligand binding, activate G proteins, leading to the regulation of cellular processes via cAMP, phospholipases, adenylyl cyclase, and Rho GTPases (7, 8). Atypical chemokine receptors, in contrast, signal independently of G proteins, primarily via β-arrestin, modulating inflammation by disrupting chemokine gradients (9).

CCR10, the last classified receptor in the CC chemokine subtype, was initially named GPR2 (G-protein coupled receptor 2) and not recognized as a chemokine receptor. Its gene, located on chromosome 17q21.1-q21.3, was identified by Marchese et al. in the 1990s (10). After the discovery of its ligand, CCL27 (ESkine/CTACK) (11), GPR2 was reclassified as CCR10 (12), with CCL28 (MEC) later identified as its second ligand (13–15).

**Figure 1:**
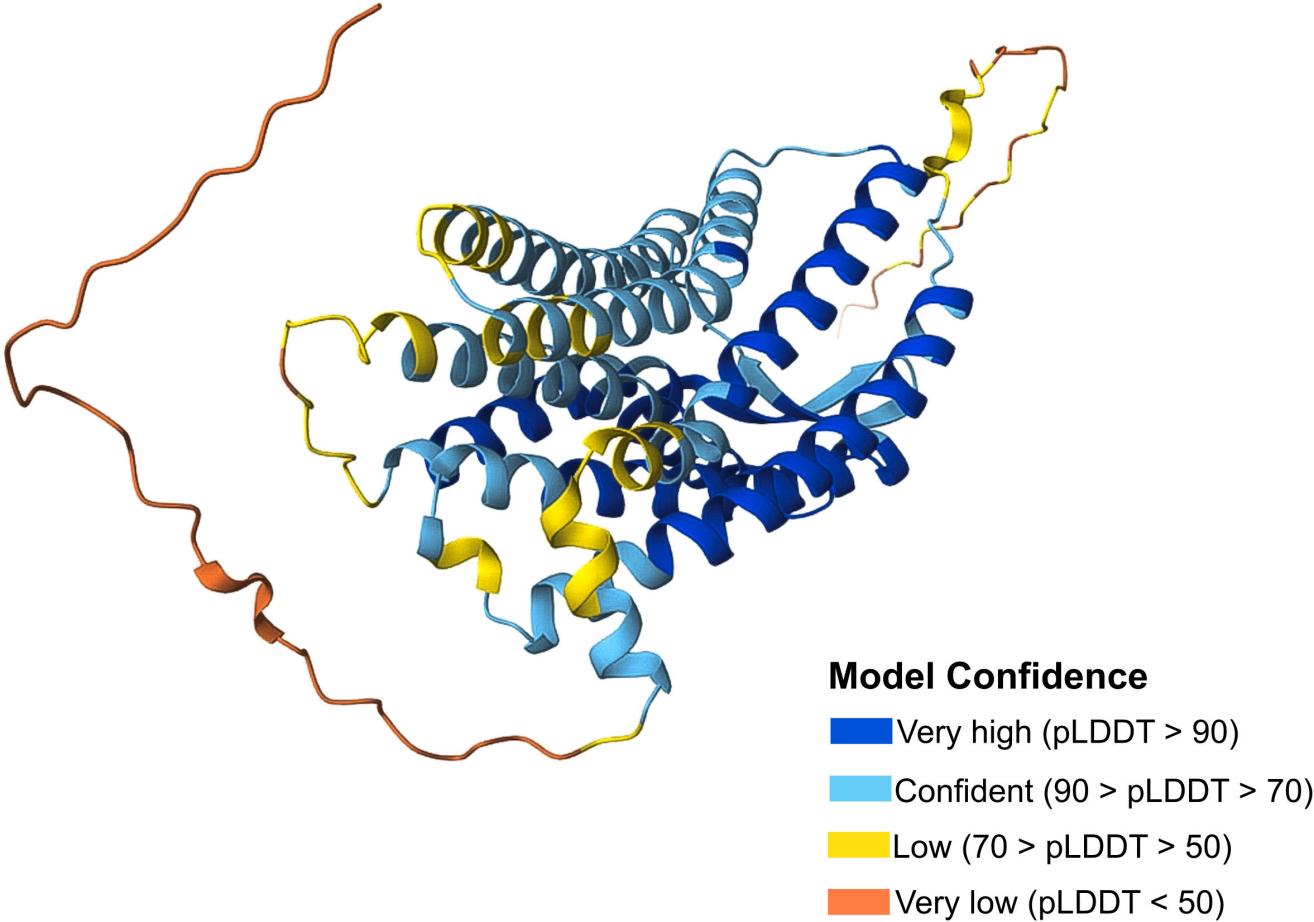
CCR10 protein structure. CCR10 Protein Structure from the Database UniProt (P46092). The confidence model of AlphaFold generates a per-residue confidence score (pLDDT) ranging from 0 to 100. Regions with low pLDDT may be unstructured in isolation. Available online: https://www.uniprot.org/.

**Figure 2:**
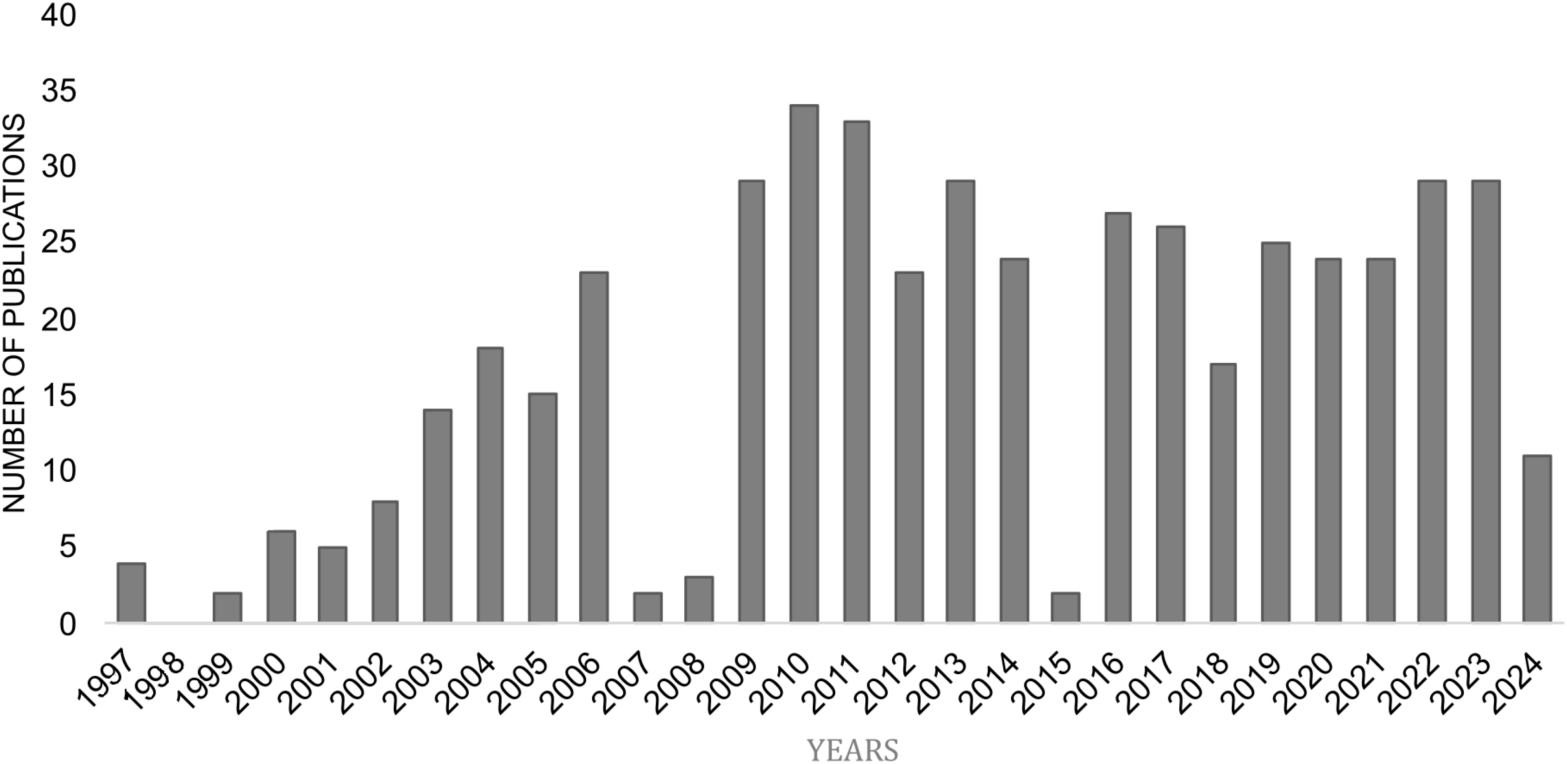
Yearly distribution of research publications on CCR10 (PubMed). Data sourced from NCBI PubMed (https://www.ncbi.nlm.nih.gov/), illustrating the annual trends in research publications focused on CCR10 from 1997 to 2024.

CCR10 consists of 362 amino acids, featuring the typical GPCR structure: an extracellular N-terminus, seven transmembrane α-helical segments, and an intracellular C-terminus (**Figure 1**) (16). Historically, CCR10 was often mistaken for the atypical chemokine receptor ACKR2/D6, which scavenges and degrades inflammatory chemokines via β-arrestin in a G protein-independent manner (17, 18). Over time, ACKR2 was correctly identified as an atypical receptor, while CCR10 was recognized as a typical chemokine receptor (19). Despite increasing scientific interest, CCR10 remains relatively understudied, with 486 indexed publications from 1997 to 2024, highlighting both its growing relevance and existing knowledge gaps (**Figure 2**). Accordingly, this review discusses CCR10’s molecular characteristics, phylogenetic background, functional roles in immune cell trafficking, and its involvement in the pathogenesis of inflammatory and neoplastic diseases, based on a systematic search of the literature.

## Materials and methods

### MESH terms

The MeSH terms used for this study included CCR10 and Cancer, CCR10 and Diseases and, CCR10 and CCL27 or CCL28. These terms were applied in the PubMed (NCBI) database (https://www.ncbi.nlm.nih.gov/) to perform a systematic search for relevant articles. The search strategy was designed to encompass studies investigating the role of CCR10 in cancer and other diseases, as well as its association with their ligands CCL27 and CCL28.

### Analysis and selection

**Figure 3:**
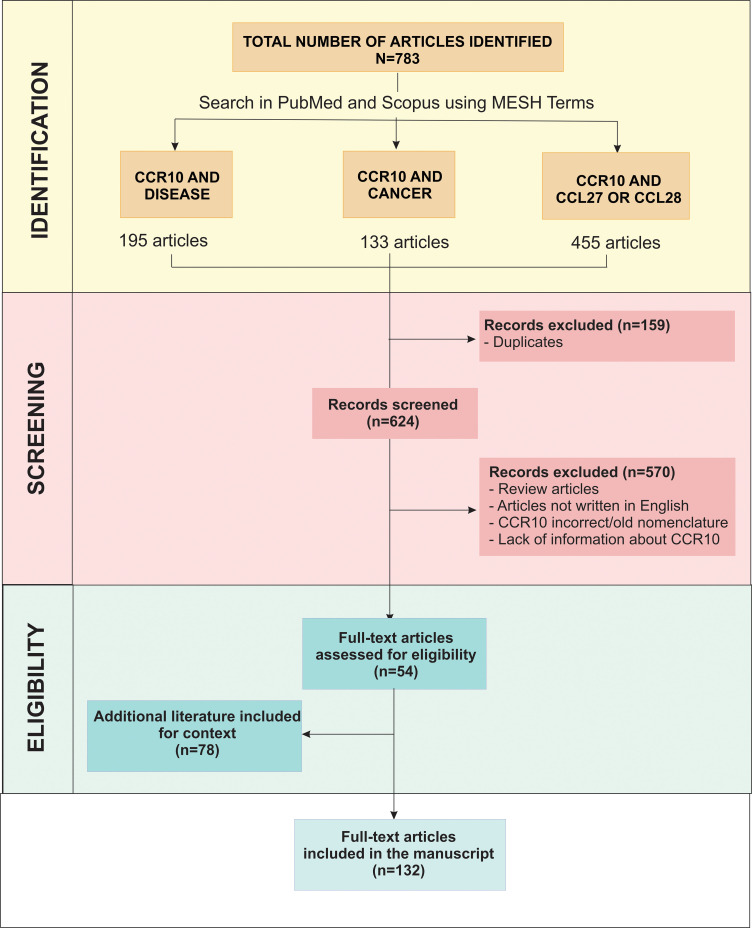
Article selection process. This flowchart illustrates the sequence of steps in selecting articles for the review, including identification, removal of duplicates, screening, and exclusion based on predefined criteria, resulting in 132 articles included in the final analysis.

We searched for articles indexed in PubMed (NCBI) and Scopus databases for the period of 1999 to 2024 using the MeSH terms “CCR10 and Disease,” “CCR10 and Cancer,” and “CCR10 and CCL27 or CCL28”, identifying a total of 783 articles. Duplicate entries found in tables generated from articles retrieved from PubMed (NCBI) using the MeSH terms “CCR10 and Disease”, “CCR10 and Cancer” and “CCR10 and CCL27 or CCL28” were unified to avoid redundancy while maintaining all relevant studies in the review. After removing 159 duplicate records, 624 articles remained for screening. During the screening phase, 570 articles were excluded as they were either review articles, translated articles, or used outdated or incorrect nomenclature, such as referring to CCR10 as D6. Additionally, studies that lacked direct information about CCR10, focusing only on its ligands without analyzing the receptor itself, were also excluded. A total of 54 full-text articles were assessed for eligibility. Additionally, 78 complementary articles were included to provide context on CCR10’s function, they were selected based on expert judgment to support the interpretation of primary studies on GPR2 and further to provide broader background on CCR10 function, chemokine signaling, immune cell trafficking and disease background. Thus, 129 articles were included in the final literature review, and the decision stages are summarized in **Figure 3**.

### Inclusion criteria

This review included only original articles written in English. The following inclusion criteria were applied:

- *In vitro* and/or *in vivo* studies investigating CCR10 under homeostatic or stimulated conditions and its impact on immune function;
- Studies analyzing the dynamics of CCR10 and its interactions with its ligands;
- Studies exploring the therapeutic potential of CCR10 in relation to various diseases;
- Additional literature offering relevant context on CCR10 mechanisms, chemokine function, or G protein-coupled receptor signaling.

### Exclusion criteria

The following exclusion criteria were applied:

- Review articles;
- Book chapters;
- Protocols;
- Editorials or expert opinions;
- Letters or communications;
- Publications in languages other than English;
- Studies focusing solely on CCR10 ligands without analyzing CCR10 itself;
- Articles using outdated or incorrect nomenclature (e.g., D6/ACKR2 instead of CCR10).

### Databases

In addition to the articles retrieved from the NCBI and Scopus databases, data regarding the expression of chemokines CCL27 and CCL28, as well as the CCR10 receptor, were collected from databases such as the Genotype-Tissue Expression (GTEx) (https://www.gtexportal.org/), UniProt (https://www.uniprot.org/) and The Human Protein Atlas (https://www.proteinatlas.org/). GTEx database was employed to analyze the expression of CCL27 and CCL28 across various tissues. UniProt was used to investigate the structural characteristics of the CCR10 receptor, while The Human Protein Atlas provided data on the expression of CCR10 in different types of immune cells. These databases were essential for complementing the analysis and ensuring a broader understanding of the topic.

### Phylogenetic tree

For the construction of the phylogenetic tree, coding sequences (CDS) of three human *CCR10* transcripts were downloaded from Ensembl (https://www.ensembl.org/). Coding sequences were selected because they represent the protein-coding regions, ensuring consistency and facilitating comparative analyses. Additionally, CDS of placental mammal orthologs were retrieved to include a broader representation of species. To root the tree, two human and one mouse *ACKR3* transcripts were chosen as outgroups. ACKR3, while a chemokine receptor like CCR10, is evolutionarily more distant, providing an appropriate level of divergence for robust tree rooting. The sequences were aligned using MEGA11 software, with the MUSCLE algorithm applied for DNA alignment. DNA alignment was chosen to maintain the integrity of the original sequences and avoid potential loss of information that could occur with protein-based alignment. The phylogenetic tree was constructed using the Maximum Likelihood (ML) method, known for its robustness and reliability in evolutionary analyses. The GTR+G evolutionary model was selected based on the lowest Bayesian Information Criterion (BIC) score (36718.231), determined using the “Find Best DNA/Protein Models” feature in MEGA11. The analysis was performed with five gamma categories, as recommended by MEGA, and included 1000 bootstrap replicates to ensure robust statistical support.

## CCR10 phylogeny and mechanisms

### Evolutionary insights into CCR10

The evolution of *CCR10* across species, highlights its pivotal role in immune response and adaptation. Diversification of the chemokine receptor superfamily has been shaped by evolutionary pressures (20), and endogenous retroviral elements within *CCR10* introns, may have influenced its regulation and trajectory.

*CCR10* contains retrovirus-like sequences, specifically Short Interspersed Nuclear Elements (SINEs), with 80% similarity to HERV-K and HERV-P-T47D, and a distinct viral insertion site with 79% identity to other sequences. Its unique Plus/Plus orientation, unlike *CCR7 and CCR9*, suggest a specific interaction with viral elements and immune evolution (21).

Co-evolutionary studies with its ligand CCL27 reveal that CCR10 remains functional even when *CCL27* is lost, as in cetaceans, which have accelerated skin renewal and reduced need for inflammatory responses. Some species, (e.g., *Delphinapterus leucas*, *Lagenorhynchus obliquidens, Hyperoodon glaber* and *Balaenoptera acutorostrata)* show incomplete *CCR10* exon 1 annotations, while *Tursiops truncatus*, lack current genome annotations, however, manual annotations confirmed an intact open reading frame (ORF) without disruptive mutations, underscoring evolutionary conservation and functional significance of CCR10 across species, despite ligand loss (22). These findings emphasize the evolutionary stability of CCR10, suggesting that it may hold importance in these species. However, its precise function remains uncertain and warrants further investigation.

**Figure 4:**
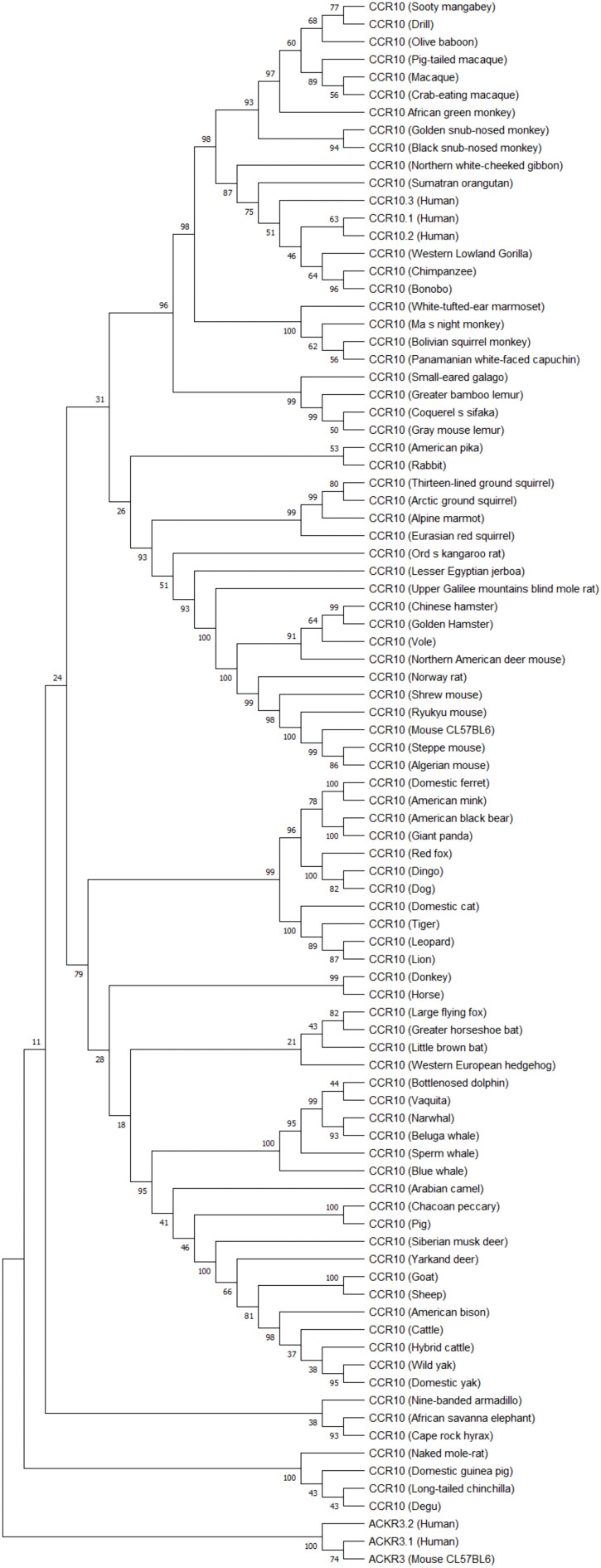
CCR10 phylogenetic tree. Coding sequences of three human CCR10 transcripts and mammalian orthologs (placental mammals) were aligned using MUSCLE in MEGA1.1. The phylogenetic tree was constructed with the Maximum Likelihood method (GTR+G model, 5 discrete gamma categories, best-fit BIC score) and rooted using ACKR3 as an outgroup due to its evolutionary distinction within the GPCR family. Bootstrap analysis (1000 replicates) assessed the tree’s robustness, highlighting CCR10’s evolutionary dynamics and potential functional insights.

Phylogenetic analysis of CCR10 (**Figure 4**), coding sequences including human variants, reveals three human isoforms (CCR10.1, CCR10.2, CCR10.3) clustering closely, with high similarity to other primates such as gorillas, chimpanzees, and orangutans, supporting functional conservation in primates.

*CCR10.3* variant exhibits greater evolutionary proximity to orangutans and gibbons compared to the other variants, suggesting it may be the oldest human variant. Beyond primates, there is a clear separation between taxonomic groups, such as rodents, carnivores, artiodactyls, perissodactyls, and cetaceans, indicating lineage-specific adaptations. Cetaceans appear most divergent group, potentially reflecting aquatic environmental pressures. The strong bootstrap supported in the phylogenetic tree confirms evolutionary CCR10 structural stability alongside with adaptive divergences over time.

### CCR10 and its ligands in immunity

**Figure 5:**
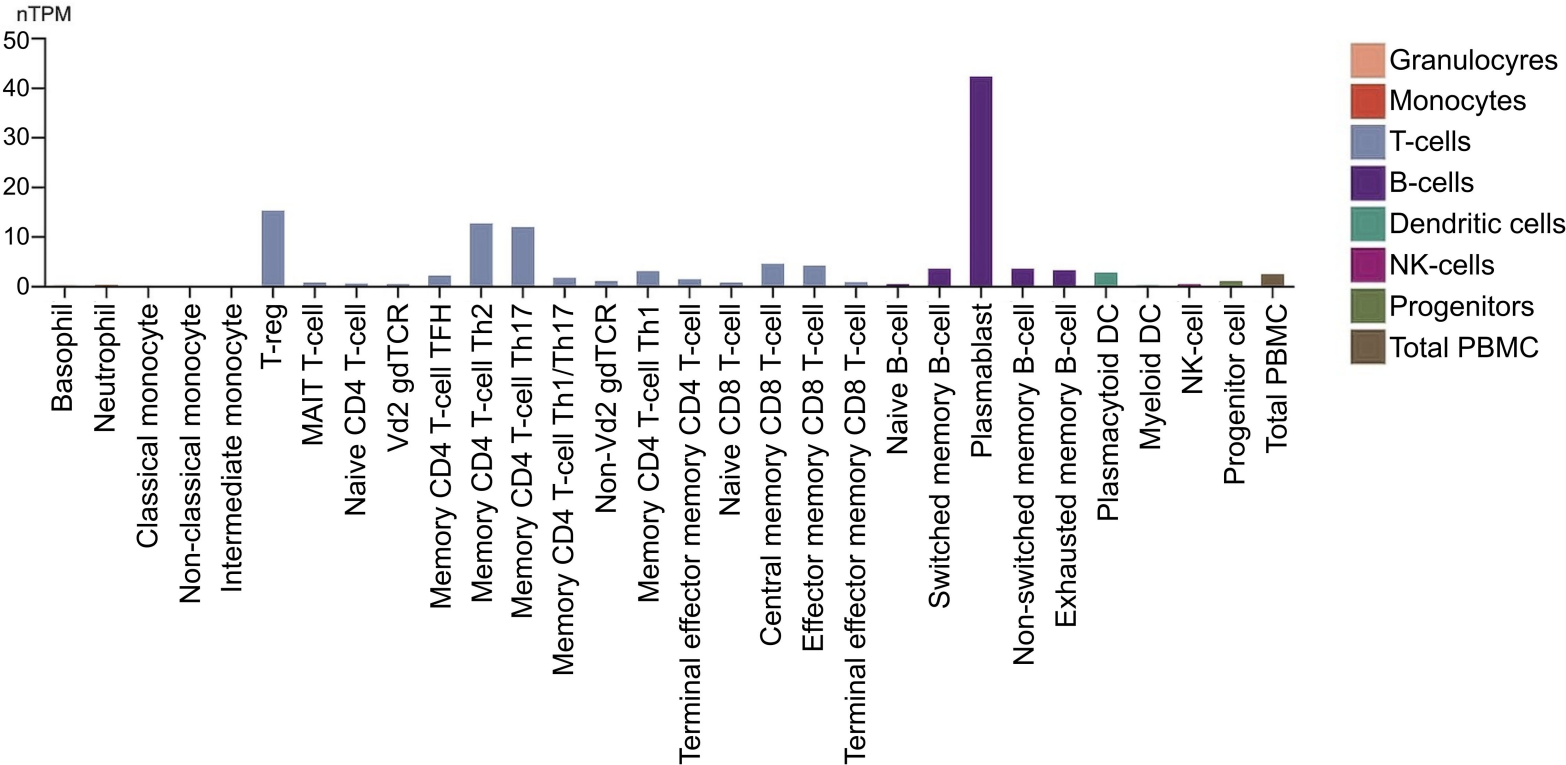
CCR10 expression in immune cells. The nTPM values were obtained through internal normalization of transcript expression, including both coding and non-coding transcripts. Expression levels are shown for 29 blood cell types and peripheral blood mononuclear cells (PBMCs), based on data from the study by Monaco et al., as presented in the Human Protein Atlas. (https://proteinatlas.org/).

CCR10 is expressed in various immune cells (**Figure 5**), predominantly in memory T cells (CD4 and CD8) in peripheral blood, particularly those with high CLA (cutaneous lymphocyte antigen) expression (23). Recent studies highlight its role in CD8 T memory cell development during skin infections, particularly in forming tissue-resident memory cells, emphasizing its function in T cell activation and memory formation (24).

CCR10 is also found in plasmacytoid dendritic cells (pDCs) and IgA plasmablasts and plasma cells (25, 26). In pDCs, it plays a key role in migration, with a distinct subset in the tonsils exhibiting functional expression. Its expression in circulating pDCs increases following IL-3 stimulation, enhancing responsiveness to CCR10-specific ligands, suggesting a mechanism for cytokine-driven migration and function (26).

Beyond immune cells, CCR10 is also expressed by dermal fibroblasts, melanocytes, and dermal microvascular endothelial cells (14).

**Figure 6:**
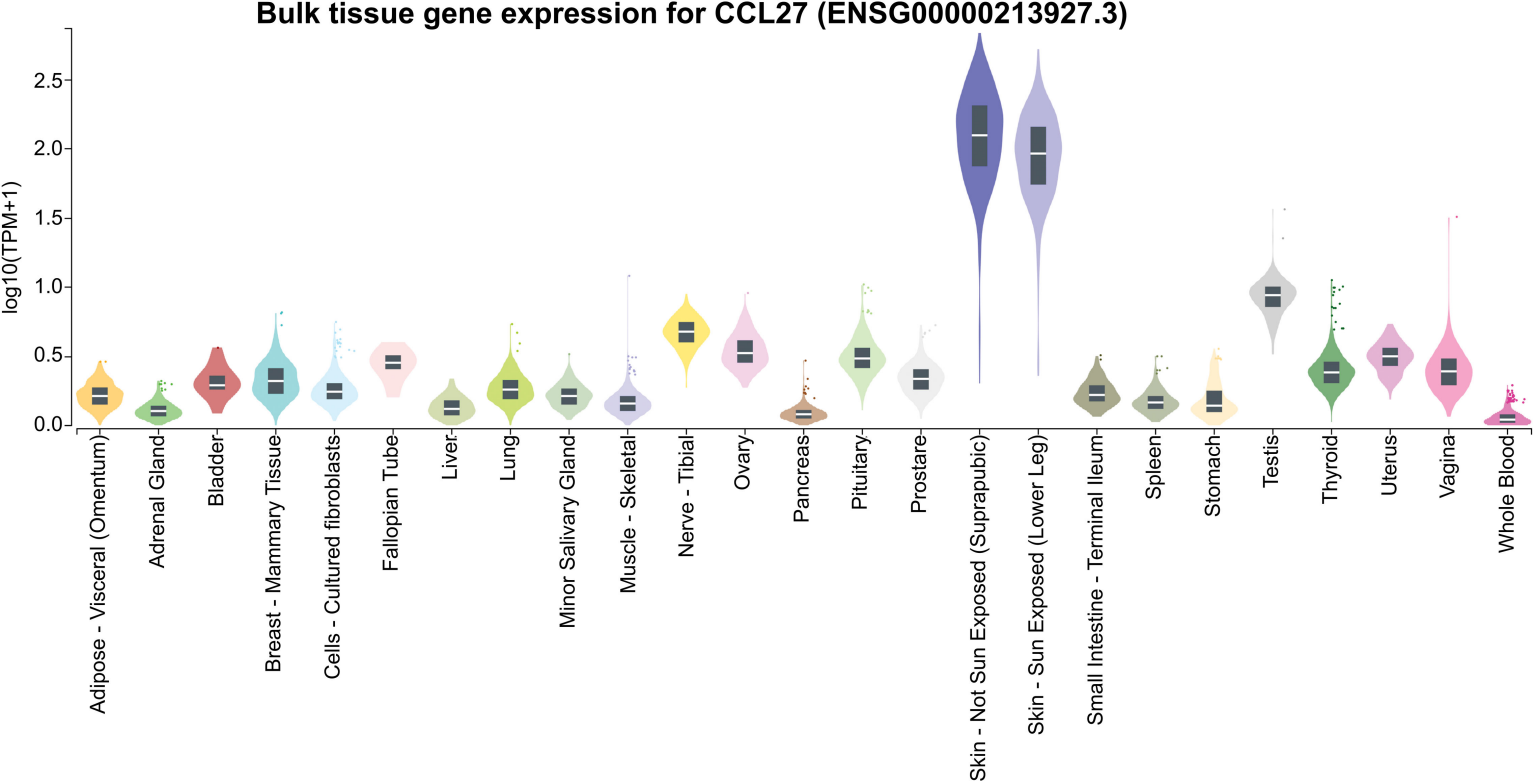
Gene expression of *CCL27* across various tissues. *CCL27* expression values are presented as TPM (Transcripts Per Million), derived from a gene model with isoforms merged into a single gene. No additional normalization was performed. Box plots represent the median and the 25th and 75th percentiles, with points displayed as outliers if they fall outside 1.5 times the interquartile range. Data obtained from GTEx (https://www.gtexportal.org/).

CCL27, the first identified ligand of CCR10, is a skin-associated homeostatic chemokine (**Figure 6**) upregulated during inflammation (27). Its expression is induced by IL-1β and TNF-α and regulated via the nuclear factor-κB (IκB) kinase (IKK) complex, MAPK, MSK1, and MNK1/2 signaling pathways (11, 28, 29). The CCL27 gene encodes multiple splice variants, including a secreted form and a non-secreted nuclear variant, PESKY (30).

After an initial CCR7-mediated recruitment from the blood to lymphoid tissues draining inflamed epithelia, pDCs may be further conditioned to express CCR6 and CCR10, thereby directing their subsequent migration toward inflamed mucosal or skin epithelia. While CCR10 facilitates pDCs migration, its chemotactic response is weaker than that induced by CXCL12, a key chemoattractant for these cells. Additionally, CCR10 expression has been linked to pDCs-mediated IFN-α secretion during viral infections, suggesting a role in antiviral immunity (26).

CCL27, mainly produced by keratinocytes, promotes tissue repair via CCR10 (27, 31), and in its neuronal isoform PESKY, is expressed in the limbic regions and cortical regions, increasing during allergic inflammation and correlating with T cell infiltration, especially in the olfactory bulb (32).

**Figure 7:**
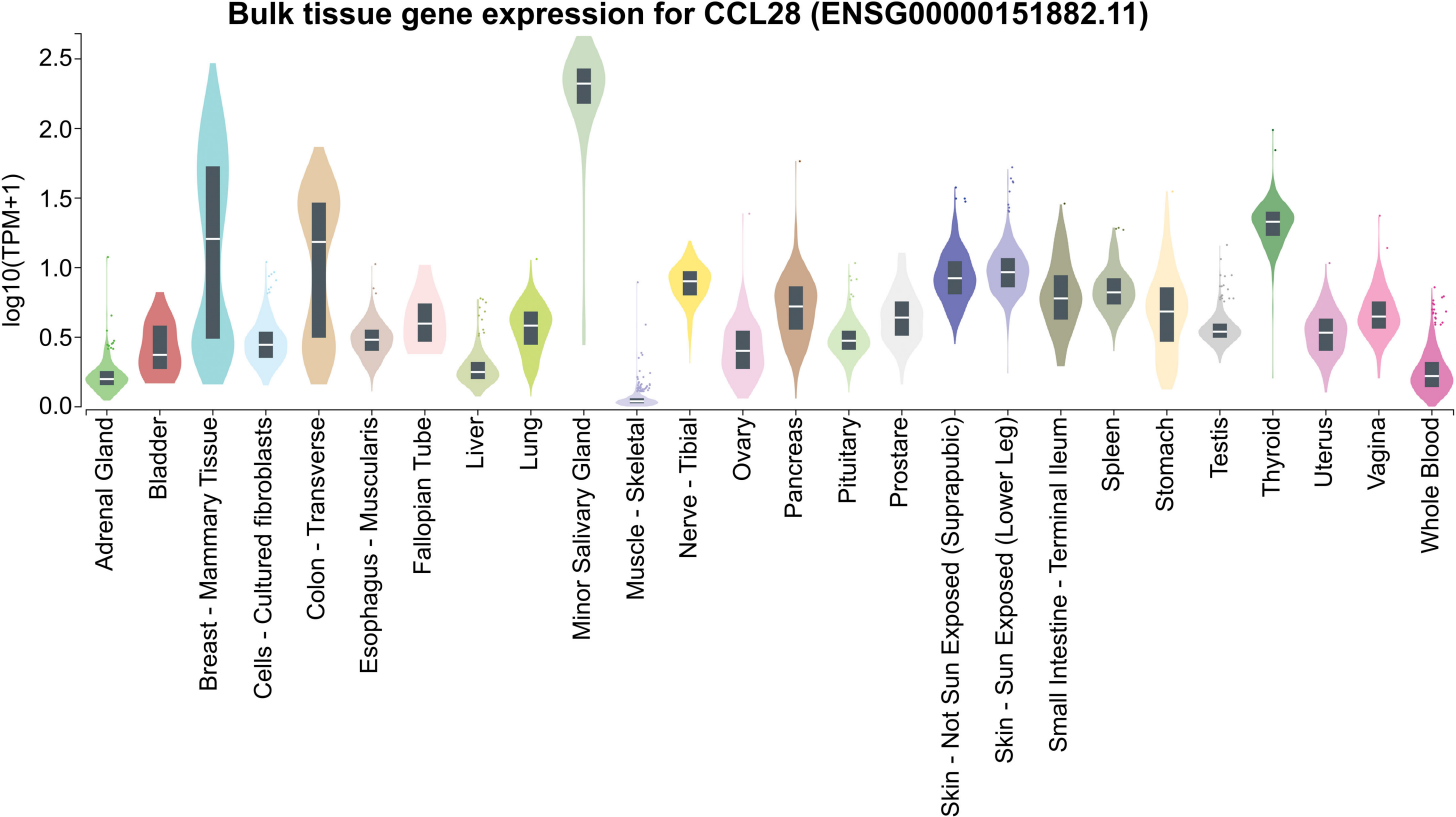
*CCL28* expression across various tissues. Expression values are presented in TPM (Transcripts Per Million), calculated from a gene model with isoforms collapsed into a single gene. No additional normalization steps were applied. Box plots show the median and the 25th and 75th percentiles; points are displayed as outliers if they are above or below 1.5 times the interquartile range. Data obtained from GTEx (https://www.gtexportal.org/).

The second ligand of CCR10, CCL28 (mucosa-associated epithelial chemokine, MEC), is expressed in salivary, mammary, and exocrine glands, as well as the intestine (**Figure 7**) (15). Its expression is induced by TNF-α and IL-1β through ERK and NF-κB signaling pathways (33). CCL28 binds both CCR10 with high affinity than CCR3 (15).

**Figure 8:**
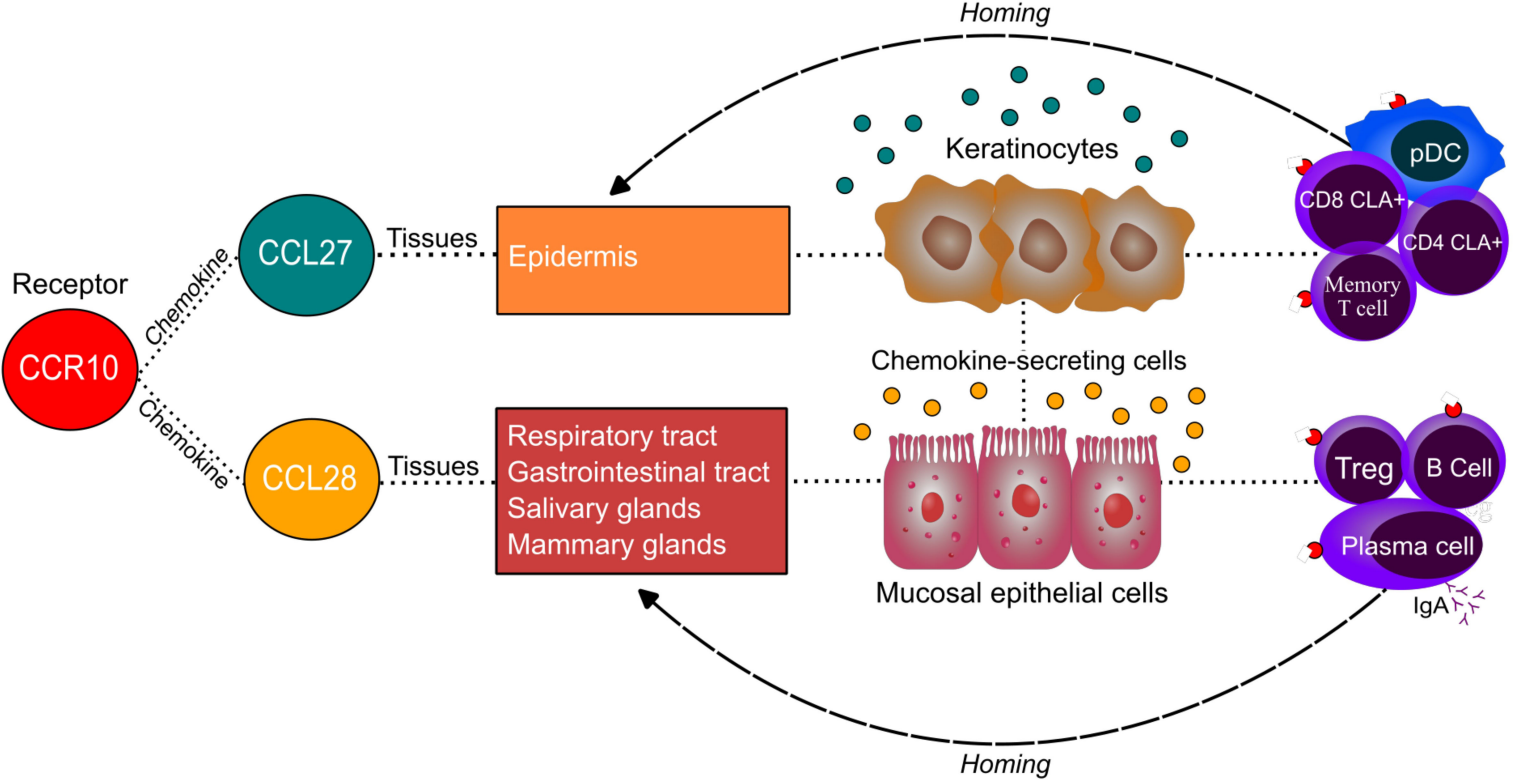
Homing of immune cells to specific tissues according to CCR10 ligands. A schematic illustration showing the interaction between the CCR10 receptor and its ligands, CCL27 and CCL28, highlighting the primary tissues where these chemokines are expressed and the cells responsible for their secretion in these tissues. The diagram also depicts the role of this interaction in lymphocyte migration to target tissues, emphasizing the immunological relevance of CCR10-mediated immune cell homing.

Secreted mainly by epithelial cells, CCL28 plays a key role in mucosal immune responses (15), linking the immune systems of the intestines, respiratory tract, and mammary glands (34). CCR10 direct IgA-secreting cells from mucosal tissues to the mammary gland during lactation (34–39), and in the intestine, CCL28 directs IgA-secreting cells to the small intestine and colon (**Figure 8**), mediating extravasation into the intestinal lamina propria (40). Beyond chemoattraction, CCL28 has antimicrobial activity against diverse pathogens, mediated by its positively charged C-terminal region (35, 39).

### Context-dependent roles of CCR10

CCR10 plays a crucial role in maintaining homeostasis and regulating inflammatory processes. However, its specific functions remain a topic of considerable debate. Initially, CCR10 was thought to primarily promote migration of T cells to both healthy and inflamed tissues (11, 12, 14, 27, 30). In individuals with skin lesions, elevated CCR10 expression has been detected in intraepidermal leukocytes, in contrast to its lower expression in healthy tissues.

Blocking the CCR10-CCL27 interaction prevents T cell recruitment to the skin in wild-type (WT) mice, while intradermal CCL27 significantly enhances T cell migration to inflamed skin (27), supporting its role in recruiting activated CD4 and CLA T cells to cutaneous tissue (27, 30), which may later return to secondary lymphoid organs to differentiate into central and effector memory T cells (23). However, some studies have suggested CCR10 is less critical for T cell migration in inflamed tissues as previously thought and instead serves homeostatic roles, including establishing skin-resident cells and regulating T regulatory cells (Treg) (41–43).

Reiss et al. showed that blocking the CCR10-CCL27 axis alone did not prevent T cell migration in WT mice but was effective in CCR4 knockout (KO) mice (44). In an oxazolone-induced allergic contact dermatitis model, CCR10 and CCR4, with their ligands, attracted tissue-resident memory T cells to inflamed skin; only simultaneous inhibition of CCL27, CCL17, or CCL22 prevent inflammation indicating redundance and compensatory roles of CCR10 and CCR4 in lymphocytes recruitment (44, 45).

Soler et al. found CCR10 expression in ~30% of skin-resident Th cells (CLA+/CCR4+), all with a memory effector phenotype. However, most skin-infiltrating lymphocytes in hypersensitivity lesions or bacterial infections expressed CCR4 and CLA, while only ~10% expressed CCR10, suggesting a specialized role in directing effector memory Th cells to epidermal microenvironments rather than broad skin homing (46).

Comparative studies in CCR4, CCR6, and CCR10 KO mice showed that only CCR4 deficiency impaired CD4 T cell accumulation in inflamed skin, while CCR10 may instead influence immune polarization (41).

In CD8 T cells, CCR10 proved important for priming and memory formation after skin infection, particularly for tissue memory resident T cells (TRM) development and survival; CCR10 expression peaked during the effector phase of skin infection and declined after 30 days (24).

Finally, CCR10 or CCL27 deficiency markedly reduced skin-resident CCR10 T cells, especially CD4 Tregs and CD8 effectors, disrupting immune cell balance and predisposing to over-reactive and prolonged innate responses (42, 47).

Loss of CCL27 diverts CCR10-expressing T away from the skin, towards lungs and reproductive tract, provoking inflammation and revealing crosstalk among barrier tissues (47).

CCR10 also shapes cytokine output and trafficking of CCR10 effectors (e.g., Th17), promoting resolution of cutaneous inflammation (42). In psoriasis models, the CCR10–CCL27 axis restrains disease: CCR10 skin ILCs arise in skin-draining lymph nodes (LNs) but decline under inflammation/homeostatic stress while CCR10 deficient ILCs expand in LNs and skin (47–49), limiting hyperactivation of IL-17A and IL-22 secreting cells as αβT, γδT, and Th17 cells, while impaired signaling exacerbates pathology (47, 48).

Beyond skin, ILC2 expressing CCR10 mitigates severe asthma via IFN-γ; their depletion plus IFN-γ blockade heightens airway hyperreactivity (50). In radiation-induced dermatitis, CCR10 supports ILC maintenance and limits inflammation, and ionizing radiation upregulates keratinocyte CCL27 via a TNF-α–ROS feedback loop (51, 52).

Together, these data highlight the CCR10–CCL27 axis as a key regulator of immune-cell trafficking and tissue homeostasis, limiting inflammation and preventing immune dysregulation in respiratory and dermatologic diseases.

### CCR10 and its ligands in tissue repair

CCR10-CCL27 promotes wound repair, primary dermal fibroblasts and dermal microvascular endothelial cells express CCR10 and show enhanced repair with CCL27. During re-epithelialization, CCL27 attracts cells such as bone marrow-derived keratinocyte precursors, and keratinocytes themselves, which express CCR10 and secrete CCL27, suggesting an autocrine loop (31, 53, 54).

The CCR10–CCL28 axis also supports repair by regulating endothelial eNOS/NO–dependent angiogenesis, LPS/IL-6 upregulate CCL28/CCR10, CCL28 activates Src–PI3K–MAPK; CCR10/eNOS binding suppresses eNOS (a key regulator of vascular tone/angiogenesis) such that disrupting this interaction or neutralizing CCL28 increases eNOS and accelerates healing, whereas CCL28 overexpression raises pro-inflammatory cytokines, CCR10, and reduces eNOS (55–58).

Beyond chemotaxis, CCR10 helps maintain skin Tregs and TRM and shapes effector polarization/localization, with the CCR10–CCL27 axis providing local immune control during injury/inflammation (**Table 1**) (27, 30, 31, 41–43, 45, 46, 53, 54). CCR10 also marks Th22 (IL-22 producing) cells, though direct links to IL-10, TGF-β, or amphiregulin production by CCR10 lymphocytes remain unproven, and its full roles in trafficking, regulation, and regeneration require further study.

**Table 1:** Mechanisms of action of CCR10 and its chemokines ligands in Inflammatory and healing processes.

CCR10 in inflammation				
↑ CCR10	↑ CD8 T cells priming	↑ Memory-resident T cells	↑ Treg	Regulated immune response
↓ CCR10	↓ CD8 T cells priming	↓ Memory-resident T cells	↓ Treg	Disregulated immune response
CCR10-CCL27/CCL28 roles in wound healing				
↑ CCL27	↑ CCR10+ Endothelial Cells/Keratinocytes/Fibroblasts			↑ Wound healing
↑ CCL28	↑ Nitric Oxide (NO)	CCR10 inhibits eNOS		↓ Wound healing

The upward arrow (↑) indicates an increase in activity or cellular response, while the downward arrow (↓) represents a decrease. The elements highlighted in green represent mechanisms or chemokines that promote healing and immune responses. In contrast, the elements marked in red indicate mechanisms that impair healing and dysregulate the immune system.

### CCR10 roles in skin and mucosal inflammation

CCR10/CCL27–CCL28 help maintain homeostasis yet can drive pathology: inflammatory dermatoses (atopic dermatitis, psoriasis, allergic contact dermatitis) show abundant CCR10 T cells and elevated CCL27/CCL28, consistent with selective Th recruitment (27, 59–63).

Conversely, CCL27/CCR10 are reduced in psoriatic lesions, alopecia areata and hidradenitis suppurativa, while a 2022 meta-analysis links higher to atopic dermatitis (AD) severity, point to disease specific regulation (64–68).

A plausible model is dynamic, context-dependent control: Th2-skewed AD upregulates CCR10/CCL27, whereas psoriasis (Th1/Th17/Th22) shifts to other chemotactic cues; notably, CCR10 marks Th22 enriched in psoriasis (62–65).

Beyond skin, CCR10 CD8 T cells are increased in psoriatic arthritis, suggesting joint/enthesis homing (69). In a keratin 14 and IL-4 AD model, keratinocyte IL-4 raised CCL27 and anti-CCL27 reduced disease (70). In allergic rhinitis, allergen exposure boosted CCR10 (NALT) and epithelial CCL28 with CCR10 memory CD4 T-cell infiltration (71).

### CCR10 and its correlation with Th22

CCR10 plays a critical role in the recruitment and function of Th22 cells in diverse inflammatory and pathological contexts, including rheumatoid arthritis (RA) (55, 72), IgA nephropathy (IgAN) (73–75), and malignant ascites (76).

In RA, both CCL28 and CCR10 are elevated in synovial tissues and fluids, where they promote angiogenesis via ERK signaling pathway (55). Th-22 cells are defined by the chemokine receptors CCR4, CCR6 and CCR10 expression and contribute to osteoclast differentiation via IL-22-mediated mechanisms. Cytokines such as TNF-α, IL-1β, and IL-6 drive their infiltration into synovial tissue in patients with active RA, where their ligands CCL17, CCL20, and CCL28 are highly expressed. Th22 cells display robust chemotaxis toward CCL28, which not only induces osteoclast formation but also enhances differentiation through IL-22, thereby linking Th22 cells activity to bone destruction character (72).

IgA nephropathy (IgAN) is a leading cause of end-stage renal disease, and Th22 cells infiltration triggered by upper respiratory tract infections, has been linked to disease progression and severity (73, 74, 77). In infection-related IgAN tubular and mesangial epithelial cells may recruit Th22 cells via CCR10-CCL27 axis, both of which are marked up-regulated in patients, particularly in tubular epithelial cells, and correlates with more severe tubulointerstitial lesions (75). *Hemolytic streptococcus* (HS) infection further amplifies CCR10 and CCL27 expression, enhancing Th22 chemotaxis and proliferation, thereby exacerbating renal injury. Blocking the CCL27 partially inhibits this recruitment, inhibiting the chemotaxis of these cells (73). Additionally, Th22 cells have been implicated in accelerating renal fibrosis in HS-related IgAN (75). Therapeutics such as Losartan and Dexamethasone significantly reduce Th22 counts and the expression of CCR10, CCL27, and IL-22, mitigating inflammation and slowing disease progression (74). Collectively, these findings highlight the CCR10-CCL27 axis as a key driver of Th22-mediated pathology in IgAN.

In malignant ascites, elevated CCL27 and infiltration of CCR10-Th22 cells observed in hepatocellular carcinoma, suggest a role for CCR10 in promoting Th22-driven pathology, although its precise function remains to be clarified (76, 78–80).

Overall, CCR10–CCL27/CCL28 interactions facilitate Th22 cell migration and activation, contributing to inflammation and positioning this axis as a potential therapeutic target in cancer and other inflammatory diseases (76, 78–80).

### CCR10 in cancer and immune regulation

S100A10 (p11) is a calcium-binding protein involved in cell proliferation, differentiation and migration, contributes to tumorigenesis, in various cancers (81–84). Forming a heterotetramer with annexin A2, S100A10 regulates plasma membrane trafficking of channels and receptors (85).

In melanoma S100A10 binds the CCR10 cytosolic tail, linking it to annexin A2 and regulating CCR10 surface expression; S100A10 knockdown increases CCR10 levels and disrupts annexin A2 association, underscoring its role in CCR10 localization and stability (86).

CCR10 also participates in tumor-associated lymph angiogenesis. Lymphatic endothelial cells (LECs) expressing CCR10, regulated by VEGF-D and TNF-α migrate towards tumor-derived CCL27 and CCL28; VEGF-D is essential for *in vivo* lymphatic vessel formation and metastasis (87–90).

In T cells malignancies, CCR10 is linked to skin infiltration. Adult T-cell leukemia and lymphoma (ATLL) patients with skin lesions display elevated CCR10 mRNA in peripheral blood and towards CCL27 and CCL28 (91).

In cutaneous T-cell lymphoma (CTCL) subtypes including Sézary Syndrome (SS), Mycosis Fungoides (MF), and Cutaneous T-Cell Lymphoma Not Otherwise Specified (CTCL-NOS), CCR10 is expressed by tumor-infiltrating lymphocytes (TILs) and malignant clones, with ~10-fold higher transcripts levels during leukemic phases and frequent chromosomal alterations involving isochromosome 17 (92, 93). In addition, MF displays elevated levels of CCR10-expressing CD4 T cells in peripheral blood and TILs, along with increased concentrations of CCL27 (94).

In SS, CCR10 expression on CLA- CD4 T cells, correlates with the epidermotropism and aggressive disease (92, 93, 95). Microarray studies show higher CCR10 in MF than ATLL cells, while CCL27 is strongly expressed in the epidermis of both diseasis (96).

Multiple myeloma plasma cell expresses high CCR10 and CCL27 levels in bone marrow, associated with poor prognosis and drug resistance. CCR10-CCL27 signaling counteracts bortezomib´s suppression of IL-10 by activating NF-κB in stromal cells; blockade of CCR10 or IL-10 reverses this resistance (97–101). CAR-T cells targeting CCR10 in a CCL27-dependent manner eliminate myeloma cells *in vitro* (98).

In classical Hodgkin lymphoma, Reed-Sternberg cells often express CCR10 and CCL28, potentially driving plasma cell recruitment and inflammatory amplification, thereby influencing disease progression (102).

In skin cancers findings are conflicting. Some studies in basal cell carcinoma (BCC) and squamous cell carcinoma (SCC) report CCL27 downregulation via Fas and epidermal growth factor receptor (EGFR) signaling to evade immunity (103). while others show elevated CCR10 and CCL27 in advanced SCC, correlating with invasion depth (104).

In melanoma CCR10 is up-regulated by TNF-α, IL-1β, and growth factors, with higher expression in node-positive cases. CCR10 and CCR7 overexpression predicts poor prognosis, and CCR10 activation by CCL27 promotes immune evasion via PI3K/Akt and lymph node metastasis (105–107). Several other studies highlight the PI3K/Akt pathway, as crucial in CCR10-mediated interactions across various cancers, including skin, liver, lung, and brain, where it contributes for cancer progression regulating tumor cell survival, proliferation, and metabolism (107–111). However, some evidence associates lower CCR10–CCL27 ratios with progression (112) or links high CCL27 with better survival (113), suggesting stage-dependent effects.

In Non-small cell lung cancer (NSCLC) signaling activates PI3K/Akt promoting VEGF-C/D, MMP-2/9, aTIMP-1/2, and NF-κB, supporting tumor growth, survival and invasion. CCR10 blockage reduces NF-κB and invasiveness. Interestingly, advanced tumors show higher CCL27 but lower CCR10 expression (109, 110, 114).

Glioblastoma overexpresses CCR10, with CCR10-CCL27-p-Akt signaling driving proliferation and invasion, blocking CCR10 or p-Akt reduces tumor growth (111).

In breast cancer CCR10 correlates with stage, capsular invasion, and nodal metastasis, with CCL27 inducing MMP-7 and ERK1/2 activation, CCR10 inhibition suppresses both (115).

Tumor-derived CCL28 recruits CCR10 Treg cells in ovarian cancer, promoting angiogenesis and immune tolerance under hypoxia (116).

In colorectal cancer, epithelial STAT3 loss increases CCL28, enhancing Treg migration, while CCL27-driven Th22 recruitment and IL-22 production, support tumor progression. Conversely, CCR10-IgA B cells are reduced, impairing mucosal immunity (80, 117–121).

In gastric cancer, β-catenin upregulates CCL28, enhancing Treg recruitment, blocking CCL28 reduces Treg infiltration and tumor growth (122).

Overall, CCR10–CCL27/CCL28 signaling exerts diverse, context-dependent roles in cancer promoting immune cell recruitment, tumor growth, invasion, angiogenesis, drug resistance, and immune evasion, while in some cases correlating with favorable outcomes. This complexity underscores the importance of disease stage, tumor type, and immune context in determining its therapeutic potential.

### CCR10 and its ligands as therapeutic target in cancer

Although CCR10 and its ligands are well established in disease pathogenesis, emerging evidence highlights their potential in cancer immunotherapy.

CCR10 can facilitate infiltration of central and effector memory T cells into tumors (24), and strategies that increase chemokine expression may boost immune responses in the tumor microenvironment. In B16BL6 melanoma model, intratumorally delivery of an adenoviral vector encoding CCL27 and other chemokines (AdRGD), enhanced activated T cell recruitment and inhibited tumor growth, optimal antitumor efficacy may be achieved when combining with systemic effector T cell activation (123).

CCR10 also mediates NK cells trafficking. In ovarian carcinoma and fibrosarcoma models. AdRGD-CCL27 promoted NK cell recruitment to tumors, but tumor regression required co-administration of AdRGD-IL-12, which resulted in more efficient antitumor activity (124).

Drugs used in multiple sclerosis treatment, such as glatiramer acetate (GA), dimethyl fumarate (DMF), and monomethyl fumarate (MMF), upregulate CCR10 expression on IL-2-stimulated NK cells, enhancing migration towards CCL27/CCL28, and boosting cytotoxic activity (125).

NK92 cells treated with DMF or MMF showed similar effects (126), and DMF directly inhibited melanoma growth and metastasis in mice (127). This mechanism may be particularly relevant in cancers with high CCR10 ligand expression, such as colorectal cancer, melanoma, and squamous cell carcinoma (125).

The CCR10-CCL28 axis supports B cell mediated antitumor immunity. In colorectal cancer, tertiary lymphoid structures (TLSs) rich in B cells correlate with better immunotherapy outcomes; CCR10-CCL28 guides plasma cells from TLSs into tumor stroma, potentially contributing to tumor control (128).

In melanoma, high CCL27 expression in the supratumoral epidermis is associated with longer tumor-free survival (103, 112, 113), whereas CCL27 downregulation in metastases and advanced SCC/BCC may represent an immune evasion strategy (103, 105).

Adoptive T cell Therapy can also harness CCR10. In 2024, Hong et al, engineered T cells to express CCR10-1G4 TCRs, which exhibited enhanced migration toward CCL28 via ERK1/2 and AKT activation and reduced tumor burdens in CCL28 overexpressing A375 melanoma xenografts (129).

Collectively, CCR10–CCL27/CCL28 signaling enhances immune cell infiltration and antitumor activity through T cells, NK cells, and plasma cells, supporting its development as a therapeutic target. However, conflicting findings regarding its role in different cancers underscore the need for further mechanistic studies to optimize CCR10-based strategies and improve clinical outcomes.

### Clinical use of CCR10/CCL27/CCL28 targeting molecules

Currently, no drugs targeting CCR10 or its ligands CCL27 and CCL28 have reached clinical use. A CCR10 antagonist, POL7085, a protein-epitope mimetic, has been shown to dose-dependently reduce allergen-induced airway hyperresponsiveness in murine models (130). Another research-only CCR10 inhibitor, BI-6901 from Boehringer Ingelheim, a potent and selective small-molecule antagonist that effectively inhibits CCL27-mediated signaling, reducing inflammation in murine contact hypersensitivity models (131). Beyond small molecules, neutralizing anti-CCL28 antibodies, when topically applied, have significantly accelerated wound healing in diabetic (db/db) mouse models by restoring eNOS expression, reducing CCR10 activation and inflammatory cytokines, increasing VEGF production, and enhancing angiogenesis (58). On the cellular therapy frontier, structure-guided CCL27-based CAR T cells targeting CCR10-expressing tumor cells, such as in multiple myeloma, have been described as promising candidates in early proof-of-concept (132).

In summary, while the CCR10/CCL27/CCL28 axis presents an appealing target for modulating immune cell trafficking and tissue responses, all efforts to date remain experimental and preclinical models, with no current human clinical trials being reported. Nonetheless, advances in molecular tools and cell engineering are now opening new avenues for translational development and clinical application.

## Conclusions

The multifaceted roles of CCR10 in immune regulation and tumor biology underscore its importance in both homeostasis and disease contexts. By guiding T cell localization to skin and mucosal tissues, CCR10 supports immune surveillance but may also be exploit by tumors to promote lymphatic dissemination and metastasis.

Its dual function demands cell type-specific and context-dependent investigation, functional studies, ideally through *in vivo* models and single-cell resolution approaches.

Emerging synthetic biology strategies such as CCR10 knock-down or overexpression, or engineering CAR T cells with CCR10-based trafficking modules offer innovative avenues to modulate immune responses and restrict tumor spread.

Insights into the temporal regulation of CCR10 during tumor evolution, combined with its conserved features across mammalian species, may inform the design of targeted immunotherapies. Deepening our understanding of CCR10 could thus unlock novel, precision-tailored interventions for immune modulation and cancer treatment.
